# Near Infrared Light Triggered Photo/Immuno-Therapy Toward Cancers

**DOI:** 10.3389/fbioe.2020.00488

**Published:** 2020-05-26

**Authors:** Xiaoxue Xu, Hongxu Lu, Ruda Lee

**Affiliations:** ^1^Institute for Biomedical Materials and Devices, Faculty of Science, University of Technology, Sydney, NSW, Australia; ^2^International Research Organization for Advanced Science and Technology, Kumamoto University, Kumamoto, Japan

**Keywords:** cancer phototherapy, near infrared light, photo/immune-therapy, photothermal therapy (PTT), photodynamic therapy, cancer

## Abstract

Nanomaterials-based phototherapies, mainly including photothermal therapy (PTT), photodynamic therapy (PDT) and photoimmunotherapy (PIT), present high efficacy, minimal invasion and negligible adverse effects in cancer treatment. The integrated phototherapeutic modalities can enhance the efficiency of cancer immunotherapy for clinical application transformation. The near-infrared (NIR) light source enables phototherapies with the high penetration depth in the biological tissues, less toxic to normal cells and tissues and a low dose of light irradiation. Mediated via the novel NIR-responsive nanomaterials, PTT and PDT are able to provoke cancer cells apoptosis from the generated heat and reactive oxygen species, respectively. The released cancer-specific antigens and membrane damage danger signals from the damaged cancer cells trigger immune responses, which would enhance the antitumor efficacy via a variety of immunotherapy. This review summarized the recent advances in NIR-triggered photo-/immune-therapeutic modalities and their synergistic mechanisms and applications toward cancers. Furthermore, the challenges, potential solutions and future directions of NIR-triggered photo-/immunotherapy were briefly discussed.

## Introduction

Cancer is still one of the top leading causes of mortality after many decades’ search and exploration for a cure in cancer from diagnosis and therapy. Currently, the available treatments to cancers in clinics are mainly surgery, chemotherapy and radiotherapy. A number of newly developed therapeutic modalities have been selectively adopted or in preliminary clinical trials including gene therapy, targeted therapy, immunotherapy, phototherapy (photothermal and photodynamic), magnetic hyperthermia therapy and other non-mainstream treatments.

The tumor cell death are caused in virtue of extrinsic stimuli in most of the aforementioned therapeutic modalities, such as chemotherapy mediated by chemotherapeutic agents, radiotherapy via various energy rays, hyperthermia therapy from the external high-frequency magnetic field, and phototherapy attributed to external light energy sources. Photothermal therapy (PTT) and photodynamic therapy (PDT) are the two representative phototherapy and are used for the tumor cells destruction through heat or reactive oxygen species respectively under irradiation of light. The external stimuli therapeutic approaches show significant advance in ablating solid primary tumors. However, the side effects from the extrinsic stimuli are quite strong and the recurrence of cancers is not controllable. Among the extrinsic stimuli, light energy in phototherapy offers lowest side effects and least systemic toxicity. Near-infrared (NIR) light is the preferable light source due to its superior tissue penetration ability. Under light irradiation, phototherapeutic agents that are little toxic in dark are turned into functional ones to selectively destroy cancer cells, without causing much damage to surrounding healthy tissues (Cheng et al., 2014).

**FIGURE 1:**
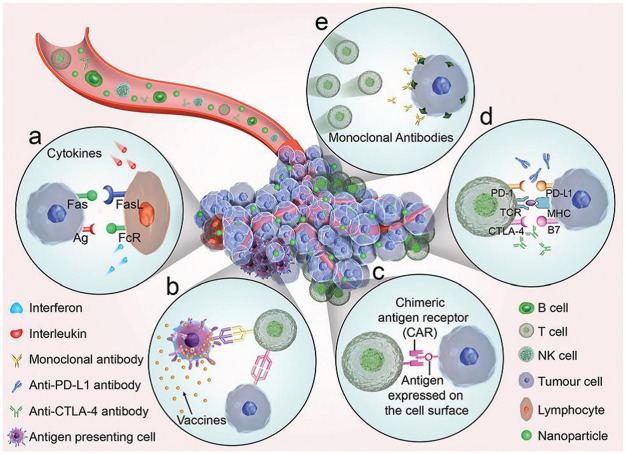
An illustrated summary of the significant cancer immunotherapeutic approaches (Sang et al., 2019). The immunotherapies function through five key components **(a)** Cytokines, **(b)** Therapeutic vaccines, **(c)** Adoptive cell transfer. **(d)** Checkpoint blockade. **(e)** Binding between antibodies and tumor antigens. Reproduced with permission from reference Sang et al. (2019).

While gene therapy and immunotherapy toward cancers are fulfilled owing to the intrinsic biological response of genes and immune systems to fight against cancers. Cancer immunotherapy approach, utilizing the patient’s own immune system to trigger or enhance the antitumor response (Galluzzi et al., 2018; Sahin and Türeci, 2018), offers several advantages over the extrinsic therapeutic methods, such as low side effects, effective treatment of metastatic cancers, and generation of immunological memory to prevent recurrence (Jo et al., 2017). The five typical types of immunotherapies to treat cancer is summarized in Figure 1 (Sang et al., 2019). Systemic administration of cytokines is the first developed immunotherapy approach. Cytokines, such as interleukins (ILs) and interferon (INF), have shown significant antitumor activity via their immune regulation effects (Smyth et al., 2004). Therapeutic cancer vaccines are developed for active immunotherapies to treat late-stage cancers by harnessing the patient’s own immune system, including peptide vaccines, whole tumor cell vaccines, dendritic cell (DC) vaccines and genetic vaccines (Guo et al., 2013). As the first Food and Drug Administration (FDA) approved therapeutic cancer vaccine, the DC-based vaccine sipuleucel-T (Provenge^®^) has been used to treat prostate cancer since 2010 (Plosker, 2011), which may drive possible success for therapeutic vaccination strategies (Hollingsworth and Jansen, 2019; Mougel et al., 2019). Adoptive T cell transfer therapy (Kalos and Carl June, 2013) is an approach that T cells with specific chimeric antigen receptors are returned back to patient’s body to eliminate cancer cells specifically after isolation, stimulation and reinfusion of tumor-infiltrating lymphocytes with potent antitumor activity. Recently, immune checkpoint pathways have entered the limelight in cancer immunotherapy because these immune checkpoint pathways control the amplitude and duration of immune responses to many cancers (Pardoll, 2012). Monoclonal antibodies, such as Ipilimumab targeting cytotoxic T lymphocyte-associated antigen 4 (CTLA-4) (Hodi et al., 2010), Atezolizumab targeting programmed death 1 (PD-1) receptor (Krishnamurthy and Jimeno, 2017), have been approved by FDA as immune checkpoint blocker for the treatments of metastatic melanoma and metastatic urothelial carcinoma or non-small-cell lung cancer, respectively. However, the immunomodulatory monoclonal antibodies have demonstrated severe immune-related adverse effects on healthy cells (Postow et al., 2015).

To achieve the ultimate goal of eradicating primary tumors and metastasizes simultaneously, utilization of synergistic therapeutic modalities with extrinsic and intrinsic mechanisms for outstanding super-additive therapeutic effects to cancers is demanding and promising in current researches (Sang et al., 2019). Extrinsic stimuli can induce immunogenic cell death (ICD) (Kroemer et al., 2013; Panzarini et al., 2013) which would release damaged-associated molecular patterns (DAMPs) thereby increase the immunogenicity of the tumor microenvironment from chemotherapy (Dudek et al., 2013), radiotherapy (Weiss et al., 2016), and phototherapy (Mroz et al., 2011). However, the resulting immune signals from the extrinsic stimuli are not sufficient and efficient to trigger the immune system for tumor erosion (Sang et al., 2019). Therefore, combining these cancer therapies with emerging immunotherapy is intuitive to overcome the limitations and maximize the merits of therapeutic modalities. From the point of view of low side effects, photoimmunotherapy (PIT), which is a synergistic therapy modality combining of phototherapy and immunotherapy toward cancers, demonstrates great potential benefits. While for the therapeutic window consideration, synergistic cancer therapy integrating phototherapy and immunotherapy would be the optimal. An optimal therapeutic window, quantified by therapeutic index, is critical to provide meaningful improvements in survival. Efforts should be aimed at increasing the therapeutic index, resulting in augmented efficacy in the elimination of primary tumor, metastasis of distant tumor and prevention of recurrence from long-term immune memory function. For the specific therapy modality, the therapeutic window can be improved from different aspects. For examples, the therapeutic index of PDT could be increased by extending the light delivery intervals (Ris et al., 1993). Exploitation of photosensitizers with low dark toxicities and high extinction coefficients may also allow the administration of higher doses of phototherapy agents and NIR light, resulting in higher therapeutic index (Boyle et al., 1992). An example is the use of Diiodo-substituted BODIPYs as a non-porphyrin photosensitizer for PDT improved the killing efficiency of melanoma B16F10 cells (Wang W. et al., 2016). Wei et al. (2019) reported a type of PIT agent, IR700-YY146, achieved high therapeutic index for melanomas of relatively smaller volumes. However, the treatment efficacy for larger tumors was not ideal due to the penetration of light through skin and tissues into the large tumor. Repeated treatment and increased the energy transfer ratio of the PIT agents may be helpful to treat large and deep tumors (Mallidi et al., 2016; Wei et al., 2019).

In this review, researches on NIR light-triggered photoimmunotherapy in last decade (2010∼2019) toward cancers using nanomaterials are collected and discussed. We first describe the therapeutic effects on cancers using the synergistic PIT of immunotherapy with PDT and PTT followed with photochemistry-based cancer therapy. Lastly, a conclusion and future perspective are drawn at the end.

## Photoimmunotherapy

Phototherapy is a non-invasive or minimally invasive therapeutic strategy. In cancer treatment, phototherapy not only kill tumor cells directly but also induces ICD to initiate a systemic antitumor immune response, including the redistribution and activation of immune effector cells, the expression and secretion of cytokines and the transformation of memory T lymphocytes (Hou et al., 2018; Li et al., 2019b).

**FIGURE 2:**
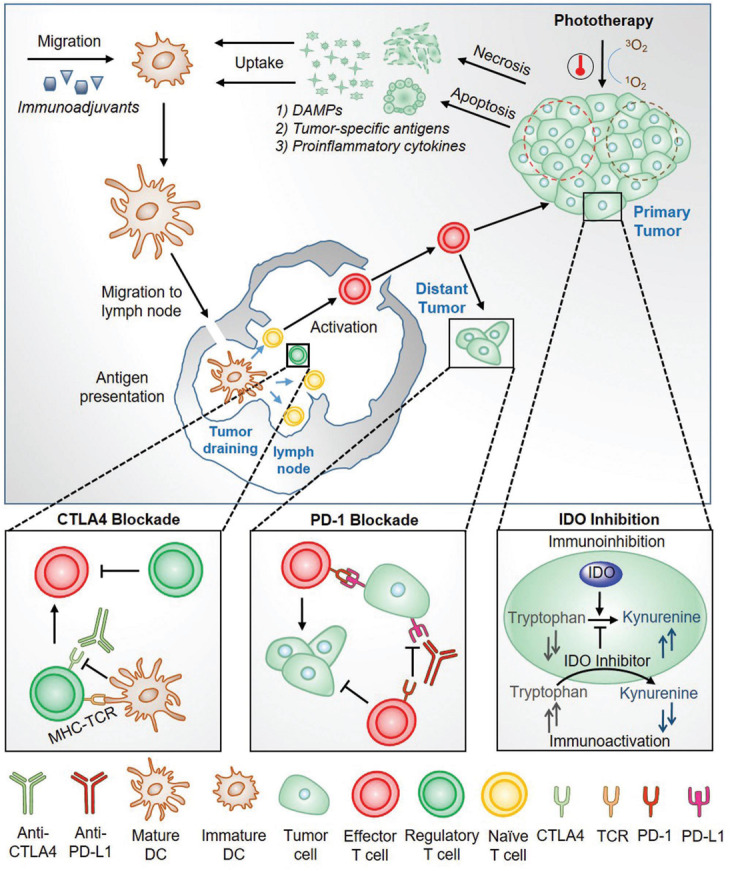
An overview of the working mechanism for the combined phototherapy and immunotherapy for cancer treatment (Ng et al., 2018). Reproduced with permission from reference Ng et al. (2018).

Combining phototherapy and immunotherapy for cancer treatment, the merits of the two will be augmented while inherent shortfalls are minimized. The general principles that allow phototherapy to greatly enhance the immunotherapy effects have been summarized by Pu’s group in a review (Ng et al., 2018) and the working mechanisms are shown in Figure 2. Firstly, phototherapy should be capable of effective eradication of primary tumors; Subsequently, the released tumor-specific antigens would serve as the substrates for an *in situ* autovaccine (Chen et al., 2016); Similarly, ICD during phototherapy would release DAMPs which are capable of triggering immune responses and thus strengthen the inherently weak immunostimulatory properties of native tumor antigens (Mroz et al., 2011); Lastly, the pro-inflammatory cytokines designed to activate the immune system are also elevated. On the other hand, immunotherapy then plays its part at the meantime with phototherapy via (1) increasing immunogenicity of the tumor microenvironment (utilizing immunoadjuvants), eventually attracting more antigen-presenting dendritic cells or (2) decreasing immunoregulatory suppression (immune checkpoint blockade therapy). In the last, the combined two therapeutic modalities could effectively eliminates the primary tumor, clears up residual tumor cells and tracks metastatic tumor sites. Phototherapy mainly is based on nanomaterials and nanotechnologies for their specific and selective effects on targeted disease lesion. Phototherapeutic modalities include PDT, PTT and photochemistry-based therapy, have attracted tremendous interest in both research and clinic aspects. The synergistic effects of each these phototherapeutic modalities with immunotherapy are discussed in the following sections respectively.

### Photothermal Therapy Synergized Immunotherapy

**FIGURE 3:**
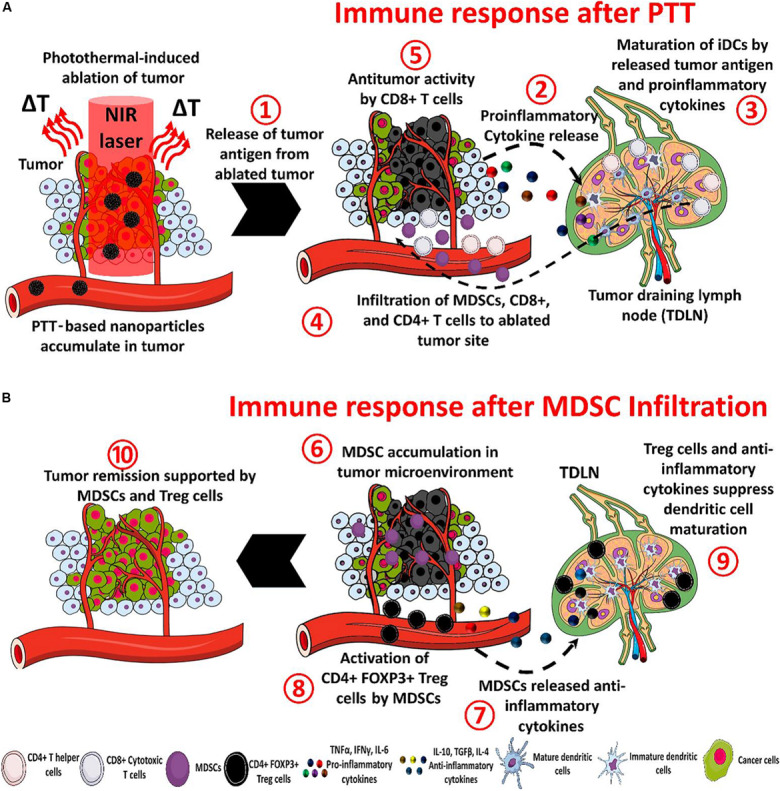
The processes of immunotherapy mediated by nanoparticle-based PTT **(A)** and the tumor recurrence. There are eight steps to treat tumors after the PTT under NIR irradiation: (1) The ablated tumor cells release antitumor antigens (2) and proinflammatory cytokines. Then (3) the cytokines and antigens promote the maturation of dendritic cells in the tumor-draining lymph node (4) and help to recruit myeloid-derived suppressor cells (MDSCs) and CD8+ and CD4+ T cells. (5) The CD8+ T cells can induce an antitumor immune response. In the tumor recurrence, (6) the MDSC infiltrate in the tumor, (7) and release anti-inflammatory cytokines, (8) CD4 + FOXP3 + Treg cells are activated by MDSCs and inhibit the antitumor immune response, (9) the maturation of dendritic cells is blocked by anti-inflammatory cytokines and activated Treg cells, and (10) the overall action of MDSCs causes tumor remission (Rajendrakumar et al., 2018). Reproduced with permission from reference Rajendrakumar et al. (2018).

PTT is one of the hyperthermia treatment to cancers through the administrated photoactive agents (Photosensitizers) that convert photon energy into thermal energy (Nam et al., 2019). Tumor cells can be killed at a temperature of 40–44°C with the invasive light irradiation, which would cause DNA damage, protein denaturation and plasma membrane disruption (Hildebrandt et al., 2002). In addition, the febrile temperature can trigger immune responses through the transcription of heat shock proteins and increased recruitment of lymphocytes to tissues with elevated temperature (Evans et al., 2015). The PTT induced tumor ablation can also induce the release of tumor antigens and immune-stimulatory molecules, thereby further activating the innate immunity and the adaptive immune system to kill residual or metastatic tumors (Hou et al., 2018). The photothermal-mediated immune response within the nanoparticles-based PTT has been illustrated in details by Rajendrakumar et al. (2018) and is shown in Figure 3.

NIR light has been widely used as the light source for PTT exhibiting minimal absorption and scattering by tissue components, thus NIR is able to achieve deep tissue penetration. Bear et al. (2013) demonstrated an effective tumor ablation strategy which adopted optically tunable gold hollow nanoshells to generate heat upon exposure to NIR radiation (808 nm). The PTT subsequently promoted the expression of pro-inflammatory cytokines (IL-6, IL-1β, IL-I2p70) and chemokines (CXCL1, CCL2, and CCL4) and induced maturation of dendritic cells within tumor-drain lymph nodes. Wang et al. (2014) also reported the photothermal ablation of primary tumors using single-walled carbon nanotubes (SWCNTs) that were able to strongly absorb NIR light of 808 nm and resulted in the release of tumor-associated antigens. However, the single model of NIR-PTT induced immune response is insufficient to control the growth of the distal tumor or metastases owing to suboptimal activation. PTT promotes the generation of immunosuppressive myeloid-derived suppressor cells (Bear et al., 2013) and induces the temperature-dependent adverse effects on cytokines and chemokines (Nam et al., 2019).

The more effective way is to integrate the two therapeutic modalities, PTT and immunotherapy, together to target the primary tumor ablation as well as the metastatic cancer cell growth. The typical immunotherapy methods have been combined with PTT via either separate but subsequent administration or single and simultaneous administration. For example, the elimination of metastatic melanoma was achieved using gold nanoshell-enabled PTT together with adoptive T cell transfer immunotherapy 24 h following PTT (Bear et al., 2013). While a cytotoxic T lymphocyte antigen-4 (CTLA-4) blockade strategy was applied into mice with breast cancer or lung cancer after the tumor ablation by PTT, the antitumor efficacy was much promoted leading to effective rejection of secondary tumors and minimized tumor metastasis (Wang et al., 2014). An interventional PTT using an optical fiber with a NIR light of 980 nm was inserted into tumor center and an immunoadjuvant agent of glycated chitosan (GC) was intratumor-injected immediately after PTT in mouse pancreatic tumor model. The complete regression was achieved and more importantly, it triggered tumor-specific immune memory and the production of memory T cells to inhibit tumor rechallenge (Zhou et al., 2018). Ma et al. (2019) adopted a second NIR (NIR II at 1086 nm) light to carry out the PTT. Compared to the red light (680 nm) and NIR I light (808 nm), NIR II light-triggered more homogeneous release and distribution of DAMPs from ICD in the deeper part of solid tumors via a gold photothermal transducer. The NIR II PTT combining with checkpoint blockade therapy enabled long-term tumor control over both primary and secondary tumors. Another NIR II transducer, polypyrrole nanosheets, showed striking therapeutic effects against whole-body tumor metastasis via the synergistic photothermal-immunological response.

**FIGURE 4:**
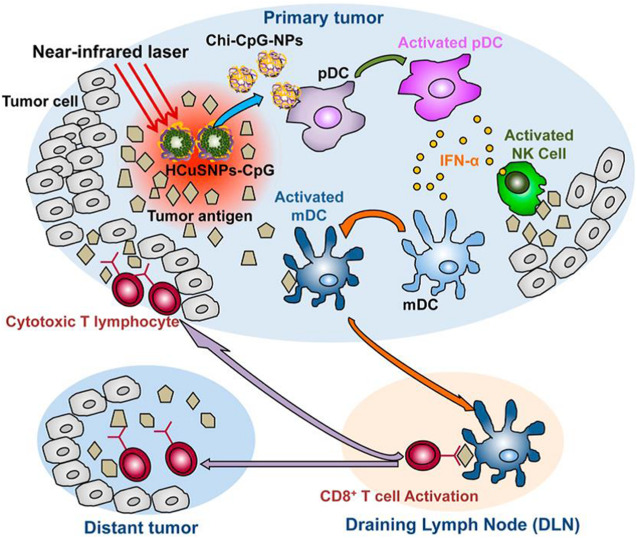
A schematic of the immunotherapy to both primary treated and distant untreated tumors mediated by chitosan-coated hollow CuS nanoparticles loaded with CpG (HCuSNPs-CpG). Upon the irradiation of 900 nm NIR light, the intratumorally injected HCuSNPs-CpG transformed to chitosan-CpG nanocomplexes (Chi-CpG-NPs) that can be internalize into Toll-like receptor 9-rich endosomes of plasmacytoid dendritic cells (pDCs). These dendritic cells produce interferon-R (IFN-R) by the stimulation with CpG, which can promote innate immunity through neutral killer (NK) cell activation. Meanwhile, the photothermal ablation by irradiated CuP nanoparticles destroys the tumor cells and releases tumor-associated antigens to attract and activate myeloid dendritic cells (mDCs). These mDCs become antigen-presenting cells with the help of IFN-R secreted by the pDCs. The antigen-presenting mDCs then migrate to tumor-draining lymph nodes (DLNs) and activate tumor antigen-specific T cells. These CD8^+^ T cells enter the systemic circulation and migrate to both primary tumor and distant tumor sites to provoke the “effector phase” of the adaptive immune response (Guo et al., 2014). Reproduced with permission from reference Rajendrakumar et al. (2018).

Guo et al. (2014) integrated the immunoadjuvant oligodeoxynucleotides containing cytosine-guanine (CpG) motifs onto the NIR light-responsive photosensitizer, chitosan-coated hollow CuS nanoparticles, and applied in a single dosage into a mouse breast cancer model. In this work, a pulsed NIR laser at 900 nm was employed. The hollow CuS absorbed the NIR light and conducted the PTT ablation to primary tumor. Upon the energy conversion, hollow CuS degraded into small nanoparticles and coated chitosan and the CpG assembled into chitosan-CpG nanocomplexes that potentiated host antitumor immunity against distant untreated tumors. The working mechanism is shown in Figure 4.

An immunoadjuvant agent, resiquimod R848, was loaded into the polydopamine nanoparticles as photothermal conversion agent together with carbon dots as imaging agent. Under 808 nm NIR light irradiation, the polydopamine can utilize hyperthermia to perform PTT to eliminate the primary 4T1 breast tumor cells. After the PTT, the generated and released tumor–associated antigens along with the PTT could be amplified by R848 and further triggered stronger infiltration of CTLs into distant tumors. In addition, The long-term immune memory effect was induced to prevent tumor reoccurrence (Lu et al., 2019).

Two different immunotherapy methods can be synergized with PTT to effectively suppress primary tumors and to inhibit metastasis. Li et al. (2019a) investigated the use of the immunoadjuvant agent, glycated Chitosan (GC), modified SWCNTs with NIR laser irradiation to potentiate anti-CTLA checkpoint blockade therapy in a highly aggressive 4T1 murine breast cancer model. The SWCNTs was adopted to achieve selective ablation of primary tumor by locally absorbing a 1064 nm NIR light and the release of tumor antigens in the tumor microenvironment, while the GC further stimulated the host immune cells and induced antitumor immune response. The combined use of anti-CTLA checkpoint blockade to suppress the activity of immunosuppressive regulatory T cells induced a systemic antitumor immunity which inhibited lung metastasis and prolonged the animal survival time.

Although the PTT induced immunotherapy efficacy is low and weak, the combined PTT with different immunotherapy has achieved substantial immune stimulation and overcome immunosuppression within the tumor microenvironment. More efforts are still required to explore synergistic and efficient PTT and immunotherapy.

### Photodynamic Therapy Synergized Immunotherapy

**FIGURE 5:**
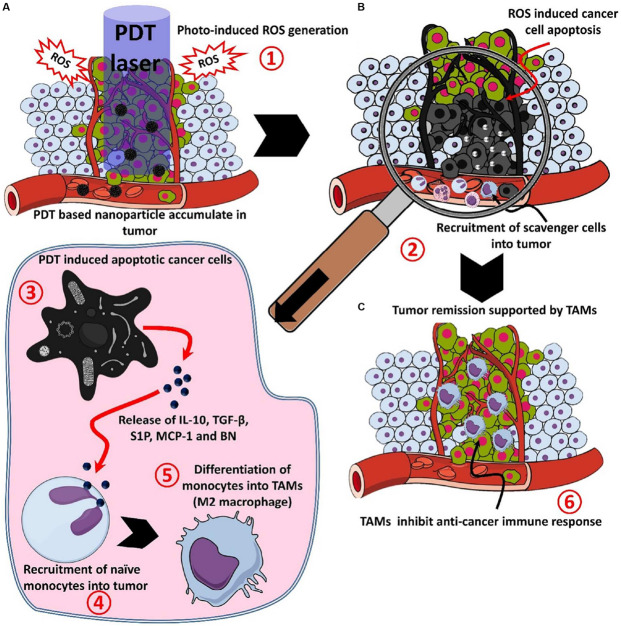
The process of the photodynamic-mediated cancer immunotherapy by nanoparticle-based PDT. **(A)** PDT laser irradiation in photosensitizer accumulated tumor leads to (1) ROS mediated cell death, **(B)** after ROS mediated cell death, (2) apoptotic or necrotic cells attracts scavenging cells like mast cells, neutrophils, and monocytes, (3) apoptotic cancer cells release factors like IL-10, TGF-β, sphingosine-1-phosphate (S1P), monocyte chemoattractant protein-1 (MCP-1) and bombesin (BN), (4) released factors attract monocytes and convert them into M2 macrophages or tumor-associated macrophages (TAMs), and **(C)** TAMs accumulated in the tumor site, (6) releases immune-suppressive proteins and cytokines to support the growth and invasion of the tumor (Xu et al., 2017). Reproduced with permission from reference Rajendrakumar et al. (2018).

PDT has been approved by the FDA for advanced cancer patients and it has been applied in clinic for various diseases with unique advantages in minimal invasiveness, low toxic side effects, good selectivity, and reproductivity (Park et al., 2018). In PDT, a photosensitizer (PS) is essentially employed to absorb the specific light energy for the generation of cytotoxic reactive oxygen species (ROS), such as singlet oxygen, H_2_O_2_ and hydroxyl and superoxide anion radicals (Chatterjee et al., 2008; Qiu et al., 2018). The ROS damages subcellular organelles and plasma membranes, thereby induce oxidative-stress-based cell death and destroy the functions of vascular surrounding the tumors. The dying tumor cells release tumor antigens and cytosolic components that provoke inflammation and stimulate immune systems. The illustration for PDT-mediated immune response following tumor necrosis after PDT is shown in Figure 5 (Rajendrakumar et al., 2018).

Similar to the PTT, PDT alone induces the relatively weak and insufficient immune response to inhibit distant metastasis while combined PDT and immunotherapy demonstrate great potential for both the primary tumor ablation and metastatic cancer cells inhibition (Wang D. et al., 2016). Compared to the hydrophobic organic photosensitizers which can selectively target in specific intracellular organelles, such as tetrapyrrole and phenothiazinium, the inorganic photosensitizers show improved PDT performances in targeted delivery and accumulation in tumor tissues and specific subcellular compartments and also in increased colloidal stability and photoresponsiveness (Abrahamse and Michael Hamblin, 2016). Single PDT using gold nanocages@MnO_2_ nanostructures as the photosensitizer was employed for enhanced PDT effect and boosted antitumor immune responses against metastatic triple-negative breast cancer. The primary problem targeted in this work was the local hypoxia tumor environment which not only severely decreased the PDT efficacy, but also caused immunosuppression by inhibiting T cells from entering tumor environment. The MnO_2_ shell degraded in the acidic (pH 6.5) tumor microenvironment and generated massive oxygen which was utilized for the production of powerful ROS from the gold nanocages under the irradiation of NIR 808 nm. The primary tumor could be destroyed by these ROS, at the same time, the immunogenic cell death–mediated antitumor immune response from DAMPs was elicited to inhibitate lung metastasis (Liang et al., 2018).

**FIGURE 6:**
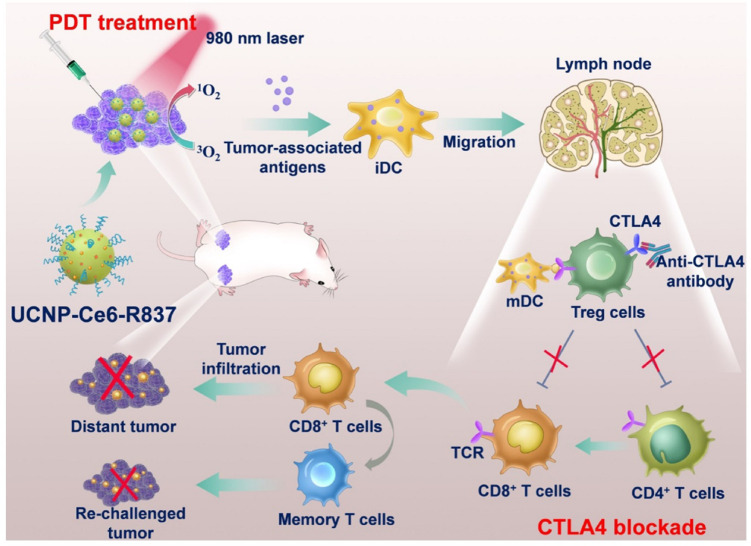
A schematic describing the mechanisms of combining NIR-mediated PDT with CTLA-4 checkpoint blockade for cancer immunotherapy. NIR irradiation to UCNP-Ce6-R837 destroys tumor cells and generate tumor-associated antigens. The R837 acts as the adjuvant to stimulate antitumor immune response. Incorporation with the CTLA-4, UCNP-Ce6-R837 can inhibit both primary and distant tumors as well as preserve a long-term immune memory to prevent tumor relapse (Xu et al., 2017). Reproduced with permission from reference Xu et al. (2017).

Upconversion nanoparticles (UCNPs) is an emerging luminescent nanomaterials with potential advantageous benefits for PDT. UCNPs are able to transfer the NIR light energy (commonly 980 or 808 nm NIR lights) into required high energy light (visible and ultraviolet lights) to excite the combined photosensitizers for the ROS generation. Xu et al. (2017) designed and constructed a multitasking UCNPs nanocomplex with a photosensitizer (Ce6) and a Toll-like-receptor-7 agonist (R837). The UCNP-Ce6-R837 nanocomplex was injected intratumorally into the tumor. Under 980 nm NIR light irradiation, the effective PDT destruction of the tumor can generate a pool of tumor-associated antigens, which was powered by the adjuvant of R837 to stimulate strong antitumor immune responses. Further combined with the checkpoint blockade immunotherapy method with CTLA-4, it has been achieved for the excellent efficacy in eliminating tumors exposed to the NIR laser and also in inhibiting the growth of distant tumors through stimulated antitumor immunities as well as long term immune memory function from reoccurrence of colorectal cancer. The working mechanism is shown in Figure 6. Lin’s group (Ding et al., 2018) designed large pore silica coated UCNPs based nanovaccine system in which photosensitizer, model protein (chicken ovalbumin, OVA) for T cell and cytokine evoke measurement and therapeutic vaccine to tumor (tumor cell fragment) could be loaded with much high loading efficiency (30 and 33.5% respectively). Both the great cellular uptake and the largely loading capacities of the prepared UCNPs–MC540 enabled much improved PDT efficacy, enhanced synergistic immunopotentiation actions to T cells and cytokines, and significant *in vivo* antitumor-therapy efficacy under 980 nm NIR irradiation.

The limitations for the PDT exist in the biosafety concern of photosensitizers, the penetration depth of the light source (NIR light can penetrate around 1 cm penetration in tissues), and less efficacy to large solid tumors. In spite of these limitations, PDT combining with immunotherapy is still a promising approach for advanced cancer treatment (Sang et al., 2019). A great deal of efforts has been contributed to develop biocompatible photosensitizers and optimize the light source type and power for penetrations in tissues and tumors.

### Photochemistry-Based Therapy Synergized Immunotherapy

**FIGURE 7:**
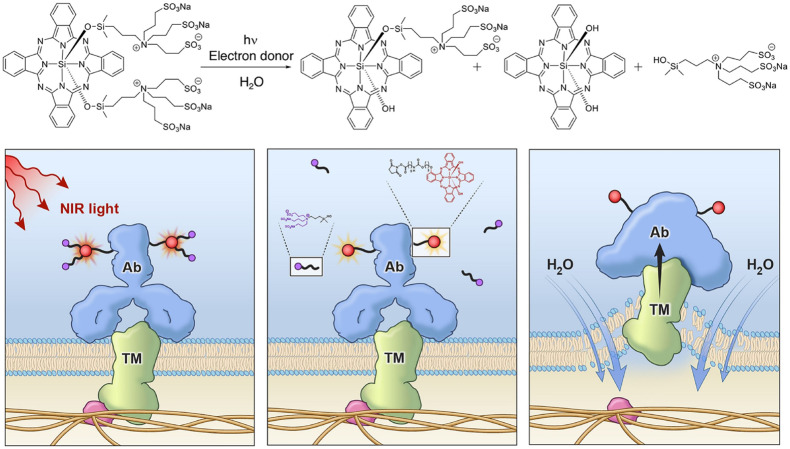
A schematic for the chemical reaction of NIR-PIT (top) and the mechanisms underlying the disruption of tumor cells (Liang et al., 2018). Reproduced with permission from reference Liang et al. (2018).

Other than the two typical phototherapy modality, PTT and PDT, NIR light is also adopted to trigger antitumor immunity as photo-immunotherapy toward cancers. NIR light was used to initiate photochemistry reactions of the photosensitizer and thus induce the immunotherapy. Kobayashi’s group (Kobayashi and Choyke, 2019) proposed a new molecularly targeted cancer photo-therapy, NIR-PIT, which is based on a NIR silica-phthalocyanine dye, IR Dye 700DX (IR700), conjugated to cell-surface molecules targeting monoclonal antibody (mAb) (Ogata et al., 2017). The water-soluble conjugated mAb with IR700 was injected into the body and the mAb targeted an expressed antigen on cancer cell surfaces. The working mechanism of the NIR-PIT is presented in Figure 7 (Sato et al., 2018). Subsequent local exposure to NIR light would photodegrade the IR700 molecules by releasing the hydrophilic side chains, which converted the IR700 molecules on the cancer cell surfaces into hydrophobic. This conversion from hydrophilicity to hydrophobicity quenched the IR700 fluorescence due to the formation of a Z-stack multimer of silicon-phthalocyanine IR700 rings or water-insoluble aggregates of antibody-photo absorber conjugate (APC) or APC-antigen complexes. The cell membrane integrity would be disrupted from the damaging of transmembrane target proteins via the physicochemical changes to the APC-antigen complex (Sato et al., 2018; Kobayashi and Choyke, 2019). Rapid swelling caused large tears in the membrane allowing the release of intracytoplasmic contents into the extracellular space (Ogawa et al., 2017). Therefore, the NIR (690 nm) was used to turn on this photochemical “death” switch to ablate the primary tumor and to induce the rapid and highly selective ICD as early as 1 min’s NIR light exposure. NIR-PIT has been demonstrated to be effective with a variety of different APCs, including anti-CD20 (Nagaya et al., 2016a) for B-cell lymphoma, prostate-specific membrane antigen (PSMA) antibody (Nagaya et al., 2017), antimesothelin antibody (Hanaoka et al., 2015) and glypican-3 targeted human heavy chain antibody for hepatocellular cancer (Nagaya et al., 2016b).

NIR-PIT was also combined with an immunotherapeutic method of checkpoint blockade (Nagaya et al., 2019). NIR-PIT monotherapy inhibited tumor growth, promoted tumor infiltration of dendritic cells, and induced *de novo* tumor antigen-specific T-cell responses absent at baseline. The addition of PD-1 blockade reversed adaptive immune resistance, resulting in both enhanced pre-existing tumor antigen-specific T-cell responses and enhanced *de novo* T-cell responses induced by NIR-PIT.

**FIGURE 8:**
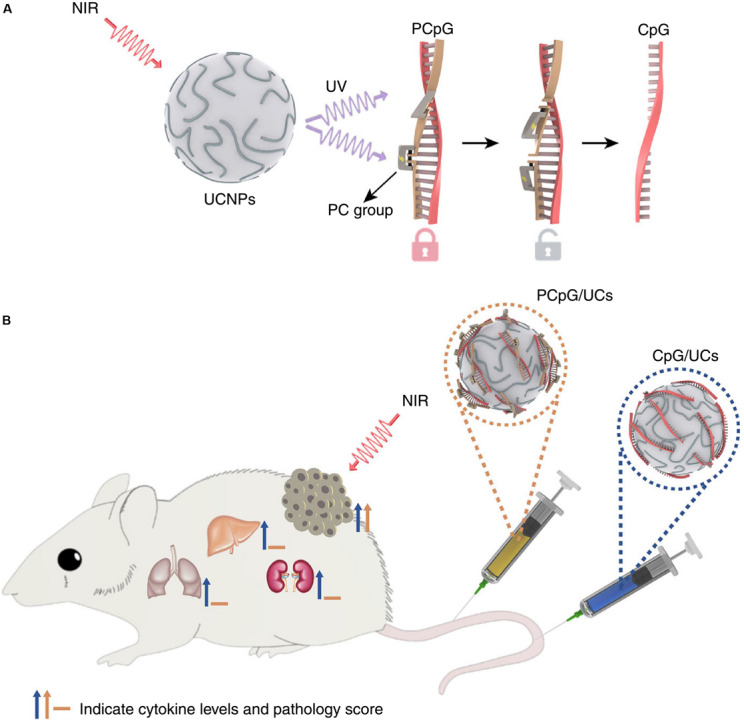
The mechanism for the antitumor effects of the photoactivatable immunodevice, PCpG/UCs **(A)** and the *in vivo* activities for PCpG/UCs **(B)**. PCpG/UCs can selectively trigger the immunoactivity through NIR light irradiation. In contrast to traditional CpG delivery system (CpG/UCs), PCpG/UCs showed reduced systemic toxicity (Chu et al., 2019). Reproduced with permission from reference Chu et al. (2019).

Chu et al. (2019) reported an activatable engineered immunodevice that enabled remotely control over the antitumor immunity *in vivo* with NIR light mediated using upconversion nanoparticles (UCNPs). It was a smart system with superior spatiotemporal precision and enhanced safety. The working mechanism scheme is displayed in Figure 8 (Chu et al., 2019). The immunodevice was composed of an immunotherapeutic agent, CpG oligonucleotides (ODNs), a complementary ssDNA (PcDNA) containing photocleavable bonds to yield PCpG, and UCNPs. The CpG was hybridized with the PcDNA firstly forming PCpG which then was integrated onto the UCNPs via cationic polymers. The constructed PCpG/UCNPs immunodevice could facilitate the delivery of PCpG into cells. After the cell uptake of the PCpG/UCNPs, upon the irradiation of NIR light (980 nm), the UCNPs converted the NIR to UV light to break the bond of the PcDNA and CpG and released the CpG inside of the cells. Thereafter, the CpG agent started to play its role for the immunotherapy to 4T1 tumor with remotely spatially selective control. This non-invasive strategy presented high spatiotemporal precision and amenable personalizing antitumor function with reduced systemic toxicity.

The photochemistry based immunotherapy shows as high potential as the PTT and PDT with less complexity in nanomaterials design and working processes. More advanced photochemistry-based immunotherapy modality should be working on.

### Multiple Phototherapy Synergized Immunotherapy

The implantation of multi-synergistic therapy relies on the combination of multiple treatments into a single nanoplatform. There are more than two phototherapeutic modalities have been integrated with immunotherapy.

**FIGURE 9:**
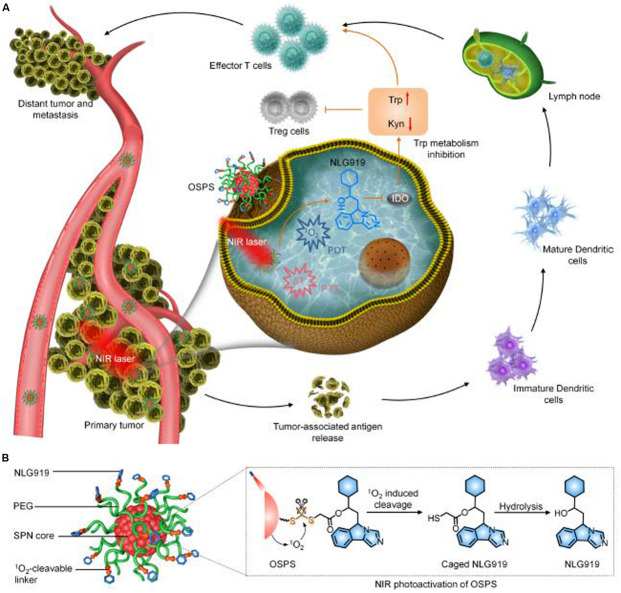
The schematic illustration of the synergistic photo/immunotherapy using OSPS nanoplatform. **(A)** Illustration of photoactivation of OSPS for synergistic therapeutic action including phototherapy and checkpoint blockade immunotherapy. **(B)** Structure and NIR photo activation mechanism of OSPS (Li et al., 2019, #45). Reproduced with permission from reference Li et al. (2019).

Synergistic therapy integrating multiple NIR photoactivatable immunotherapeutic strategy has been comprehensively investigated. Li et al. (2019) developed an organic semiconducting pro-nanostimulants (OSPS) platform for synergetic cancer therapy. The OSPS comprised a semiconducting polymer nanoparticle (SPN) core and an immune checkpoint inhibitor (NLG919) which was conjugated together by a singlet oxygen (^1^O_2_) cleavable linker. Upon NIR laser irradiation, the SPN core within OSPS could generate both heat and ^1^O_2_ for combinational PTT and PDT of tumors, leading to the release of tumor-associated antigens. Meanwhile, the generated ^1^O_2_ specifically cleaves the ^1^O_2_-cleavable liner to trigger the on-demand release and simultaneous activation of caged NLG919, which resulted in the promoted proliferation and activation of effector T cell but suppression of Treg cells. Therefore, the synergistic phototherapy with remote-controlled immune checkpoint blockade therapy exerted an amplified therapeutic efficacy in inhibition of primary and distant tumor growth and lung metastasis (Li et al., 2019b) and the working mechanism is shown in Figure 9.

Targeting for the rational design of smart nanomaterial that could combine multimodal therapy and overcome their own inherent limitations, Chen et al. (2019) developed a multifunctional nanoplatform for synergistic phototherapy (PDT and PTT), and chemotherapy drug docetaxel (DTX) enhanced cytokine immunotherapy. Mesoporous CuS nanoparticles were adopted as the carrier for loading and delivering chemotherapy drug docetaxel (DTX) and PTT agent under 808 nm light irradiation. Folic acid molecules were modified onto the CuS for targeted delivery and accumulation to tumor site. The photosensitizer, polyethylenimine-protoporphyrin IX (PEI-PpIX), were then coated outside of the CuS nanoparticle and the CpG was further modified onto the CuS surface. The combination of the four therapeutic modalities in this nanocomplex was denoted as FA-CD@PP-CpG. Although the FA-CD@PP-CpG presented remarkably inhabitation to tumor growth without apparent side effects within a 4T1-tumor-bearing mouse model, it also showed the complicated conduction for individual therapies, such as the PDT and PTT under 650 nm 808 nm light irradiation respectively, chemotherapeutic drug and immunoadjuvent agent separately release. The synergistic effect could be improved.

Yan et al. (2019) composed a relatively simple hybrid nanosystem using polydopamine as core, UCNPs nanoparticles as the shell and the photosensitizer chlorin e6 (Ce6) as surface coating. Polydopamine could absorb the excitation 980 nm NIR light and also the emissions of visible light from UCNPs for PTT. The visible light emission from UCNPs under NIR excitation was able to excite the Ce6 for PDT. The specific core-shell nanostructure design allowed adequate photoabsorption for the heat conversion and ROS generation with NIR light irradiation. Incorporating with immune checkpoint blocked-based therapy, this nanosystem inhibited tumor recurrence and metastasis and extended the survival periods of tumor-bearing mice in metastatic mouse models (Yan et al., 2019). However, the relatively large size of the core-shell nanostructure might not be able to have high accumulative amount in tumor site when systematically administrated in living mice model.

To increase the immune response induced by phototherapy, an NIR-triggered antigen-capturing nanoplatform was designed and fabricated, using UCNPs as carrier, indocyanine green (ICG) as a light absorber, rose bengal (RB) as photosensitizer and the lipid molecule (DSPE-PEG-maleimide) as the antigen-capturing agent. The ICG significantly enhanced the RB-based PDT efficiency of UCNP/ICG/RB-mal under NIR irradiation, simultaneously achieving selective PTT effects. Most importantly, tumor-derived antigens released from the ablated tumor could be captured and retained *in situ* due to the functionality of maleimide. It would further enhance the tumor antigen uptake and recruitment of antigen-presenting cells, such as dendritic cells. The synergized photothermal, photodynamic, and immunological effects from NIR-activated UCNP/ICG/RB-mal induced tumor-specific immune responses and eliminated the primary distant tumors in using a 4T1-tumor mouse model (Wang et al., 2019).

A cascade bioreactor for synergistic cancer therapy combining chemo-dynamic therapy (CDT)/starvation therapy/phototherapy/immunotherapy was developed based on hollow structured Cu_2_MoS_4_ nanoparticles loaded with glucose oxidase (GOx) (Chang et al., 2019). The hollow Cu_2_MoS_4_ was well capable of chemical dynamic therapy from the generation of the hydroxyl radicals (OH) through the chemical reactions of the redox couples of Cu^1+^/^2+^ and Mo^4+^/^6+^. The radicals also could deplete the overexpressed glutathione to modify the tumor microenvironment aiming for the mitigation of antioxidant capability of the tumors.

Furthermore, the Cu_2_MoS_4_ could catalyze the reaction of endogenous H_2_O_2_ to O_2_ which would react with glucose and produce H_2_O_2_ again under the catalysis of GOx. The consummation of glucose could shut down nutrient supply to cancer cells and achieve starvation therapy. At the same time, the phototherapy effect of the Cu_2_MoS_4_ under 1064 nm NIR light irradiation was demonstrated by the strong tumor-killing capacity due to its excellent photothermal conversion efficiency. More importantly, the PEGylated Cu_2_MoS_4_@GOx-based synergistic therapy combined with checkpoint blockade therapy could elicite robust immune responses for both effectively ablating primary tumors and inhibiting cancer metastasis.

The aforementioned photo/immunotherapy are summarized in Table 1 including the PDT synergized immunotherapy, PTT synergized immunotherapy, photochemistry based therapy synergized immunotherapy and multiple phototherapy synergized immunotherapy.

**TABLE 1:** Summary of near infrared light triggered photoimmuno-therapy toward cancers.

**Phototherapy Model**	**Immunotherapy Model**	**Mediated Nanomaterials**	**NIR light source**	**Targeted Cancer**	**Effectiveness**	**References**
PTT	Adoptive cell transfer	Gold nanoshells	808 nm	Metastatic Melanoma	− Elimination of metastatic melanoma	Bear et al., 2013
	Checkpoint blockade	PEGylated SWNTs	808 nm	Lung metastasis model	− Effective rejection of secondary tumors − Minimized tumor metastasis	Wang et al., 2014
	Therapeutic vaccines	Optical fiber	980 nm	Pancreatic tumor model	− Complete regression of primary tumor − Triggered tumor-specific immune memory and production of memory T cells	Zhou et al., 2018
	Checkpoint blockade	Gold nanoparticles	1086 nm	Breast cancer model	− Long-term tumor control over both primary and secondary tumors	Ma et al., 2019
		Polypyrrole nanosheets			− Striking therapeutic effects against whole-body tumor metastasis	
	Therapeutic vaccines	Chitosan-coated hollow CuS nanoparticles	900 nm	Breast cancer model	− Combined anticancer effects against primary treated as well as distant untreated tumors	Guo et al., 2014
	Checkpoint blockade	Polydopamine-carbon dots	808 nm	Breast cancer model	− Ablation of the primary tumor − Amplified stronger infiltration of CTLs into distant tumors	Lu et al., 2019
	Checkpoint blockade	SWCNTs	1064 nm	4T1 murine breast cancer model	− Effectively suppression on primary tumors − Inhabitation of metastatic cancers	Li et al., 2019a
PDT	-	Au nanocages @MnO_2_	808 nm	Metastatic triple-negative breast cancer	− *In situ* oxygenation to ameliorate the hypoxia environment in tumor area − High PDT efficacy and elicited antitumor immune response − Elimination of primary tumor and inhabited lung metastasis	Liang et al., 2018
	Checkpoint blockade	UCNPs + Chlorin e6	980 nm	Mouse colon adenocarcinoma (CT26)	− Enhanced tissue penetration depth thus effective photodynamic destruction of tumors − The generated a pool of tumor-associated antigens, together with CTLA4 resulted in strong antitumor immunities to inhibit the growth of distant tumors − A long-term immune memory function to protect treated mice from tumor cell rechallenge	Xu et al., 2017
	vaccine	UCNPs@large pore silica + merocyanine 540 + OVA/TF agent	980 nm	Mouse colon adenocarcinoma	− Largely load photosensitizer and immune antigens − Great cellular uptake − Enhanced immunotherapy efficacy and new approach to advanced vaccine delivery system for cancer therapy	Ding et al., 2018
PCT	Monoclonal antibodies	Silica-phthalocyanine dye (IR700)	690 nm	B-cell lymphoma, prostate cancer, hepatocellular cancer	− Photosensitizer molecule was conjugated to monoclonal antibodies − Photochemistry therapy destructed the primary tumor within 1 min under NIR irradiation	Hanaoka et al., 2015; Nagaya et al., 2016a, b, 2017; Ogawa et al., 2017; Sato et al., 2018
	Checkpoint blockade	Silica-phthalocyanine dye (IR700) conjugated with anti-CD44	690 nm	Colon and lung cancer	− Elimination of primary tumor − Durable antitumor immunity eradicated both treated and distant untreated tumors	Nagaya et al., 2019
	Cytokine	UCNPs/CpG	980 nm	Mouse 4T1 breast cancer	− Immunotherapeutic agents delivered into cancer cells and released upon the remote NIR light irradiation.	Chu et al., 2019
PDT + PTT	Checkpoint blockade	Organic semidonducting pro-nanostimulants	808 nm (0.3 W/cm^2^)	Mouse 4T1 breast cancer	− With the small size (26 nm) and stealthy PEG surface, OSPS could effectively accumulate into the tumors of living mice after systemic administration. − Upon the 808 nm light irradiation, PTT and PDT ablated the primary tumor. − ^1^O_2_ also cleaved the conjugated immunostimulant and inhibited the growth of both primary and distant tumors and suppressed lung metastasis. − Low *in vivo* toxicity.	Li et al., 2019
PTT + PDT + Chemotherapy	Checkpoint blockade	Mesoporous CuS + PEI-PpIX + DTX + CpG	808 nm and 650 nm	Mouse 4T1 breast cancer	− Negligible toxicity to normal tissues − Remarkable damage to tumors *in vivo*	Chen et al., 2019
PTT + PDT	Checkpoint blockade	Polydopamine + UCNPs + Ce6	980 nm	Mouse 4T1 breast cancer	− Significant eradication of the primary tumors from PDT and PTT. − Effective delay to untreated distal tumor from the combined checkpoint blockade therapy.	Yan et al., 2019
PTT + CDT + starvation therapy	Checkpoint blockade	PEGylated Cu_2_MoS_4_@GOx	1064 nm (0.48 W/cm^2^)	Mouse cervix cancer and lung metastasis model	− Great biosafety of the combined treatment approach. − Effective primary tumor ablation from PTT, CDT and starvation therapy; − Inhabitation of distant tumor and lung metastasis.	Chang et al., 2019
PTT + PDT	Checkpoint blockade	UCNPs + ICG + RB + DSPE-PEG-maleimide	805 nm	Mouse 4T1 breast cancer	− Efficient destruction of primary tumor; − Inhabitation of metastasis by simultaneously boosting specific immune response and checkpoint blockade. − Strong long-term antitumor immune memory function.	Wang et al., 2019

## Perspectives and Conclusion

Challenges and promising solutions are ahead of the success of photo/immunotherapy in cancer treatment. One of the major challenges is the lack of clinical trial results. Preclinical research in animals has shown that the photoimmunotherapy can eliminate tumor cells and enhance tumor-cell-selective systemic host-immunity leading to boosted antitumor effects triggered by NIR light. However, due to the species difference and complexity of human *in vivo* microenvironment, the performance of photoimmunotherapy in human is barely predicted. For example, Sato et al. (2014) compared the performance of two types of photoimmunotherapy agents, cetuximab-IR700 and panitumumab-IR700, in EGFR positive mouse tumor models. Although panitumumab-IR700 showed better antitumor efficiency than cetuximab-IR700 in the mice models, it is not possible to conclude their performance in human bodies (Sato et al., 2014). Luckily, a NIR-PIT using cetuximab-IR700 (RM1929) targeting EGFR in inoperable recurrent head and neck cancer is in a phase III trial, which is currently underway in several countries in Asia, North America, and Europe (Kobayashi and Choyke, 2019). It is expected that more photoimmunotherapy agents will be tested in clinical trials and the results will bring more valuable information for immunotherapy research. In addition to the clinical trials, the recent progress in organoids and organ-on-chips research may also provide tools for the exploitation of photoimmunotherapy (Yin et al., 2016; Brassard and Lutolf, 2019).

Functional targeting moieties and targeting efficiency is another critical factor that would influence the localized treatment. The targeting moieties bind surface markers on the cancer cells, dominating the specificity and distribution of the photoimmunotherapy agents. Various targeting moieties including antibodies, peptides, functional molecules and oligonucleotides have been conjugated with the photoactivation complexes (Kobayashi and Choyke, 2019; Shirasu et al., 2019). More specific targeting moieties will be the key to improve the efficiency of eliminating cancer cells while to reduce the side effects to the healthy cells. Targeting moieties for circulating and metastatic cancer cells are also highly desired in order to promote the prognosis after photoimmunotherapy treatment. The recent advances have applied multiple targeting groups in the photoimmunotherapy agents, aiming for cancer cells and immunocytes, respectively. The stroma cells, such as cancer-associated fibroblasts, may also be considered as a target in the photoimmunotherapy treatment (Shirasu et al., 2019).

The nanomaterials provide a large pool of candidates as the photoactivated cores for photoimmunotherapy. The development of nanomaterials with low cytotoxicity, high photoactivity, and multifunctionality will facilitate the clinical application of cancer photoimmunotherapy. Proper NIR light is required for deep penetration into the tumour tissues, dependent on the nanomaterials applied in the therapy. The energy of NIR light dramatically decreases with the depth of the tissue, for example, NIR photoimmunotherapy through bone is limited to 3 mm when using 810 nm NIR of less than 1 W (Nakamura et al., 2019). Improving the energy transfer efficiency of nanomaterials is critical for the treatment of cancers in the deep body with NIR in the biosafety range. An example is polydopamine coating improved the absorption capacity of gold nanoparticles, reported by Nam et al. (2019). It has been reported that IR700-formed APC showed a biodistribution similar to non-conjugated parental antibodies, once injected intravenously, the APC will have a sufficient accumulation in the tumors (Mitsunaga et al., 2011; Nagaya et al., 2018). However, it is still challenging for the nanomaterials-based phototherapy that the recognization and clearance of the nanomaterials by the cells in liver, spleen and other parts of the reticuloendothelial system (RES) may reduce the concentration of the therapeutics in the tumor sites. The intratumoral route of administration are generally chosen to overcome the RES uptake and low time blood circulation. In addition, nanomateriasl are often modified with PEG (PEGylation) (Gao et al., 2020) or zwitterionic polymers (Zheng et al., 2019) to hide the nanomaterials from the RES cells and to enhance the accumulation at tumors.

Well-integrated photo/immunotherapeutic modalities will benefit the single administration and working simultaneously. PTT and PDT, in combination with immunotherapies, provide infinite possibilities as a promising method to treat cancer. The administration or conjugation of antibodies or gene silencing agents aiming to immune checkpoint pathways with PTT and PDT open new windows for immunotherapy (Wang W. et al., 2016). However, the performance and efficiency of integrated strategies are not always comparable in terms of eradication of primary tumors, inconsistent results between different models, and difficulty in conjugating biomarkers. More comprehensive studies with integrated therapies will benefit the development of successful photoimmunotherapy agents.

The ultimate goal of the nanomaterials-based phototherapies is to eradicate cancers completely, including primary solid tumors and metastatic cancers. With minimized side effect and maximized treatment efficacy, phototherapy and immunotherapy are the two most promising therapeutic modalities and their synergistic combination of photoimmunotherapy has shown substantial potentials to realize the ultimate goal.

## Author Contributions

XX designed, structured, and wrote the manuscript. HL designed, structured, and revised the manuscript. RL designed andrevised the manuscript.

## Conflict of Interest

The authors declare that the research was conducted in the absence of any commercial or financial relationships that could be construed as a potential conflict of interest.
